# Frequency and power dependence of the sonochemical reaction

**DOI:** 10.1016/j.ultsonch.2021.105858

**Published:** 2021-12-03

**Authors:** Yoshiyuki Asakura, Keiji Yasuda

**Affiliations:** a Honda Electronics Co., Ltd., Toyohashi, Aichi 441-3193, Japan; b Department of Chemical Systems Engineering, Graduate School of Engineering, Nagoya University, Nagoya, Aichi 464-8603, Japan

**Keywords:** Reaction rate, Ultrasonic power, Ultrasonic frequency, Quenching

## Abstract

The dependence of the sonochemical reaction on ultrasonic intensity was studied over a wide frequency range of 22–1960 kHz and sample volume range of 25–200 mL. The effect of a stainless steel reflector set on the water surface was also considered. Experiments were carried out by direct ultrasonic irradiation of a sample in a vessel. The potassium iodide (KI) method was used to evaluate the sonochemical reaction in terms of efficiency and reaction rate, and calorimetry was used to determine ultrasonic power. A quenching phenomenon, where the reaction rate decreased despite an increasing ultrasonic power, was observed at all frequencies and sample volumes, which indicated the existence of a maximum reaction rate. The maximum reaction rate increased with the frequency, except at 1960 kHz, and with the sample volume. The ultrasonic power at which quenching occurred increased with the frequency and sample volume. Sudden quenching occurred without the reflector, whereas gradual quenching occurred with the reflector. Based on the results, ultrasonic power density (i.e., ultrasonic power divided by the sample volume) can be used to estimate the ultrasonic power at which quenching occurs for various sample volumes.

## Highlights

• Dependence of sonochemical reaction on the ultrasonic power studied at 22–1960 kHz.
• KI method used to measure the sonochemical reaction for sample volumes of 25–200 mL.
• Reaction quenching was observed at all frequencies and sample volumes.
• Ultrasonic power at which quenching occurred increased with frequency.
• Maximum reaction rate increased with the sample volume.

## 1 Introduction

When water is irradiated with ultrasound, fine bubbles are generated from bubble nuclei and repeatedly expand and contract. The fine bubbles collapse after they grow to a certain size by rectified diffusion and bubble–bubble coalescence [1], [2]. Because the collapse of the fine bubbles by ultrasonic irradiation is caused by semiadiabatic compression, the field inside the fine bubbles reaches a high temperature and high pressure. This generates various radical species in and near the fine bubbles, which produces chemical effects [3]. The local reaction field where fine bubbles collapse because of ultrasonic irradiation is called a hot spot, and the series of phenomena (i.e., fine bubble generation, growth, and collapse) is called ultrasonic cavitation. Physical effects such as a jet flow and shock waves also occur in hot spots and near collapsing bubbles [4]. Therefore, ultrasonic cavitation produces both chemical and physical effects. Examples of the physical effects of ultrasonic cavitation include ultrasonic cleaning [5], [6], ultrasonic emulsification [7], [8], [9], and ultrasonic atomization [10], [11]. These have been used to develop commercial products such as ultrasonic cleaners, ultrasonic homogenizers, and ultrasonic atomizers. Examples of the chemical effects of ultrasonic cavitation include the decomposition of harmful substances [12] and the synthesis of metal nanoparticles [13]. However, these reactions have been performed only at a laboratory scale. Because a large volume, short processing time, and high yield are required for commercialization, not many products that utilize the chemical effects of ultrasonic irradiation have been developed. However, many studies have reported interesting results, such as the decomposition of harmful substances without oxidizing agents [12], [14] and the synthesis of metal nanoparticles without reducing agents [15]. Such results are interesting because these chemical effects are caused by radical species that are produced solely by water pyrolysis under ultrasonic irradiation. Therefore, sonochemistry (i.e., chemistry by ultrasound) is expected to realize industrial application soon.

Increasing the reaction rate is a key factor for the industrial application of sonochemistry. The sonochemical reaction rate depends on various factors such as the physical properties of the sample [16], [17], [18], [19], [20], ultrasonic frequency [21], [22], [23], [24], amount and type of dissolved gas [25], [26], [27], and ultrasonic intensity [28]. Optimizing ultrasonic frequency and intensity are important for increasing the sonochemical reaction rate. In addition, an approach to quantify the sonochemical reaction rate is required.

Mason et al. [29] used fluorescence to quantify hydroxy terephthalate ions at frequencies of 20, 38, 40, and 60 kHz. They used calorimetry to measure the ultrasonic energy in samples and reported that the chemical effect was greater at 60 kHz. Koda et al. [30] used Fricke, potassium iodide (KI), and TPPS (porphyrin derivatives) dosimetry to measure the sonochemical reaction rate and calorimetry to measure the ultrasonic power for a sample volume of 50 mL and frequency range of 19.5–1200 kHz. They defined sonochemical efficiency as the ratio of the amount of reacted substance to ultrasonic energy and reported that sonochemical efficiency was the highest at 200–500 kHz. The authors [31] previously investigated sonochemical efficiency by varying liquid height at frequencies of 45, 128, 231, and 490 kHz, and the results showed that sonochemical efficiency depended on liquid height at each frequency. For example, highest sonochemical efficiency at 45 kHz was obtained at 500 mm of liquid height. However, the effect of a wide range of ultrasonic intensities on sonochemical efficiency has not yet been reported.

As the ultrasound intensity increases, the reaction rate increases due to an increase in the number of cavitation bubbles and an increase in the temperature within the cavitation bubbles [30], [32], [33], [34]. However, the reaction rate greatly decreases when ultrasonic intensity becomes quite high. This phenomenon is called quenching. Negishi [35] investigated the relationship between ultrasonic intensity and sonoluminescence at 470 kHz. Sonoluminescence increased with ultrasonic intensity and reached a maximum before decreasing. Berlan et al. [36] studied the effect of the voltage applied to a transducer and reported that the reaction rate increased with the applied voltage until it reached a maximum and then decreased significantly at higher applied voltages. Mitome et al. [37] irradiated water with pulsed ultrasound at 43.7 and 130 kHz to measure the quenching of sonoluminescence. Hatanaka et al. [28], [38] used photomultiplier tubes at 23, 44, 99, and 132.2 kHz to investigate the dependence of the sonoluminescence of multiple bubbles on the electric power applied to the transducers. They reported that sonoluminescence increased with the applied electric power and then decreased because of quenching and that the electric power at the onset of quenching increased with frequency. However, because previous studies evaluated quenching using the voltage or electric power applied to a transducer, the results are inapplicable to ultrasonic devices with transducers and vessels of differing shapes and sizes.

The ultrasonic intensity at which quenching occurs is important for sonochemistry experiments and industrialization. To increase the sonochemical reaction rate, ultrasonic intensity needs to be increased in the range where quenching does not occur. In this study, the ultrasonic energy applied to water per unit time was defined as the ultrasonic power. The advantage of using ultrasonic power is that the results are applicable to devices with transducers and vessels of various shapes and sizes.

In this study, the dependence of the sonochemical efficiency on ultrasonic power was investigated for a wide frequency range. The ultrasonic power at which quenching occurs was identified, and the ultrasonic power range in which quenching does not occur was determined. The relationship between ultrasonic power and sonochemical reaction rate was investigated for different sample volumes to evaluate the dependence of the ultrasonic power at which quenching occurs on the volume.

## 2 Experimental

### 2.1 Apparatus

**Fig. 1:**
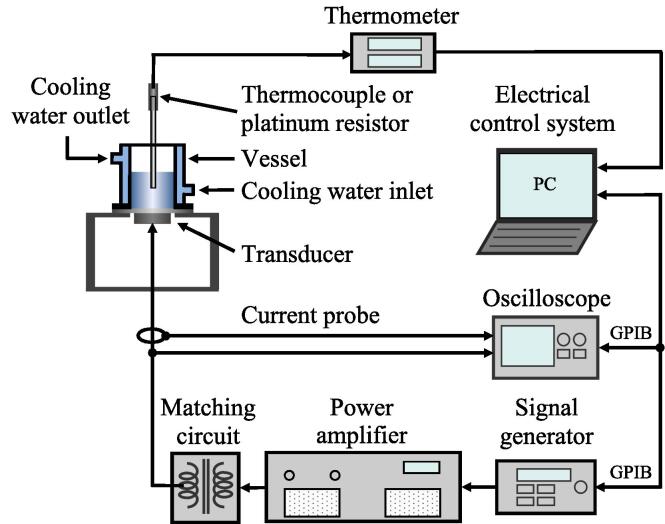
Experimental setup.

Fig. 1 shows the experimental setup. The vessel had an inner diameter of 56 mm and a double-layer structure to circulate cooling water. A vibration plate with a transducer was attached to the bottom of the vessel. The vessel and vibration plate were made of SUS304 stainless steel. A Langevin-type multifrequency transducer (HEC45242M, Honda Electronics) with a 45 mm diameter was used at frequencies of 22, 43, 97, and 129 kHz, and a disk-type transducer (Honda Electronics) with a 50 mm diameter was used at frequencies of 209, 305, 400, 514, 1018, and 1960 kHz. The transducers were driven by a power amplifier (1040L, E&I) that amplified a continuous sine wave generated by a signal generator (WF1942, NF). Effective electric power was calculated from the voltage measured at both ends of the transducer by an oscilloscope (TDS3014B, Tektronix), and the current flowing through the transducer was measured by a current probe (TCP202, Tektronix).

An electrical control system (Honda Electronics) was used to maintain a constant effective electric power. The system read the effective electric power measured by the oscilloscope, which was sent to a personal computer via a general-purpose interface bus (GPIB). Then, it optimized the output voltage for the signal generator, and a command was sent from the personal computer to the signal generator via the GPIB. A matching circuit (Honda Electronics) was inserted between the power amplifier and transducer, except for measurements at 305, 400, and 514 kHz.

### 2.2 Measurements

#### 2.2.1 Ultrasonic power

Calorimetry was used to determine ultrasonic power (i.e., energy applied to a sample per unit time). The temperature of the sample in the vessel was measured using a thermocouple (T-type, Takahashi Thermo) or platinum resistor (pt100, Netsushin) and a thermometer (NR500, Keyence). Ultrapure water (Milli-Q Reference & Elix Essential UV5, Merck) was used as the sample. Ultrasonic power *P*_U_ was calculated as follows:

(1) $$P_{U}=\frac{\Delta T}{\Delta t}C_{p}M$$

where Δ*T/*Δ*t* is the rate of the temperature rise, *C*_p_ is the specific heat capacity of water, and *M* is the mass of water. The rate of the temperature rise was determined from the change in temperature in the initial stage of ultrasonic irradiation or before and after ultrasonic irradiation [39]. The sample volumes were 25, 50, 100, 150, and 200 mL. The sample temperature before ultrasonic irradiation was 298 ± 0.5 K, and the sample was saturated with air. During the measurement of the water temperature, the cooling water was not circulated. The ultrasonic irradiation time was 120 s for all experiments.

#### 2.2.2 KI oxidation

The KI method was used to determine the chemical effect. When ultrasound is irradiated into an aqueous KI solution, I^−^ ions are oxidized to give I_2_. When excess I^−^ ions are present in solutions, I_2_ reacts with the excess I^−^ ion to form I_3_^−^ ion as follows:

(2) $$I_{2}+I_{}^{-}\;⇄\;I_{3}^{-}$$

KI aqueous solution was prepared with KI (Fujifilm Wako Pure Chemicals) and ultrapure water at a concentration of 0.1 M and in volumes of 25, 50, 100, 150, and 200 mL. The I_3_^−^ concentration was measured at 352 nm using an ultraviolet spectrometer (UV-1850, Shimadzu Corporation) and quartz cuvette. Sonochemical efficiency (SE) [mol·J^−1^] was used to evaluate the chemical effects of ultrasound [30], and it can be defined as

(3) $$SE=\frac{\mathrm{AV}}{P_{U}\varepsilon lt}$$

where *A* is the absorbance of I_3_^−^ [−], *V* is solution volume [L], *P*_U_ is ultrasonic power [W], *ε* is the molar extinction coefficient of I_3_^−^ [L·mol^−1^·cm^−1^], *l* is cuvette length [cm], and *t* is sonication time [s]. In this experiment, *ε*, *l*, and *t* were 26,303 L·mol^−1^·cm^−1^, 1 cm, and 120 s, respectively. The sample before ultrasonic irradiation was saturated with air at a temperature of 298 ± 0.1 K. The I_3_^−^ reaction rate *k*_I3_ [mol·s^−1^] was calculated as follows:

(4) $$k_{I3}=\frac{\mathrm{AV}}{\varepsilon lt}=SE∙P_{U}$$

## 3 Results and discussion

### 3.1 Ultrasonic power

**Fig. 2:**
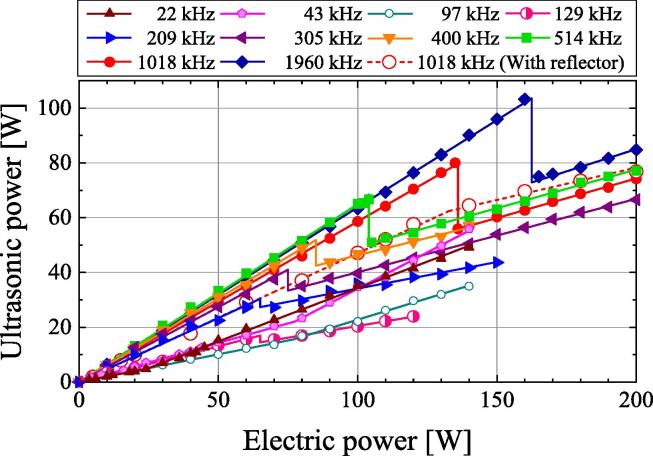
Relationship between ultrasonic power according to calorimetry and electric power.

Fig. 2 shows the relationship between the effective electric power applied to the transducer and the ultrasonic power at various frequencies. The ultrasound is irradiated upward from the transducer. The sample surface is free without the reflector, and the sample volume is 100 mL. In the frequency range of 129–1960 kHz, the ultrasonic power increases linearly with the effective electric power. However, the ultrasonic power decreases significantly at effective electric powers of 136 and 162 W at frequencies of 1018 and 1960 kHz, respectively, before increasing again with the effective electric power. A sudden decrease in ultrasonic power is observed in the frequency range of 129–1960 kHz. The effective electric power at which the ultrasonic power decreases suddenly and the magnitude of the sudden decrease in ultrasonic power are greater at higher frequencies.

Fig. 2 also shows the relationship between the effective electric power and ultrasonic power when measured at a frequency of 1018 kHz and with a stainless steel reflector on the water surface. When the effective electric power is less than 135 W, the ultrasonic power is lower with the reflector than without the reflector. With the reflector, the ultrasonic power increases with the effective electric power, and no sudden decrease in ultrasonic power is observed in contrast to the case without the reflector.

At frequencies below 129 kHz, the ultrasonic power increases with the effective electric power. The relationship between the effective electric power and ultrasonic power becomes nonlinear. The refraction point of the effective electric powers are 25, 78, and 77 W at frequencies of 22, 43, and 97 kHz, respectively. At higher powers, the gradient of the ultrasonic power to the effective electric power increases. At frequencies of 22 and 43 kHz, many cavitation bubbles occur near the transducer, audible sounds are heard, and bubbles are observed. This increases the heat generated by the vibration of many bubbles, which increases the gradient of the ultrasonic power to the effective electric power.

### 3.2 Evaluation with the KI method

#### 3.2.1 Dependence of sonochemical efficiency on ultrasonic power and frequency

**Fig. 3:**
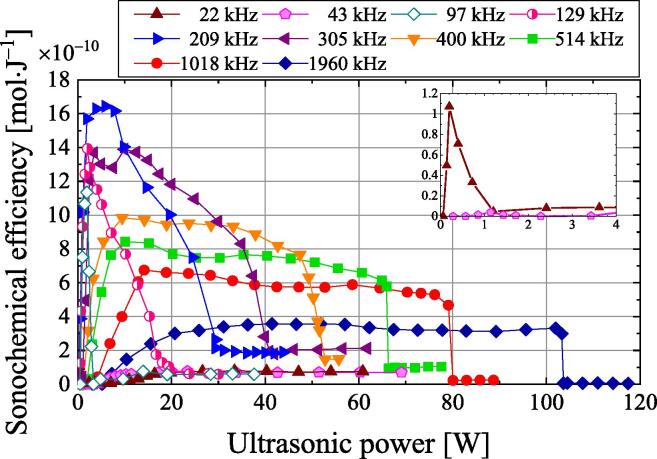
Dependence of sonochemical efficiency on ultrasonic power at various frequencies (inset: magnified sonochemical efficiencies at 22 and 43 kHz).

Fig. 3 shows the dependence of the SE on the ultrasonic power at various frequencies. The inset magnifies the sonochemical efficiencies at frequencies of 22 and 43 kHz. The sample volume is 100 mL. At frequencies of 22, 43, 97, 129, and 209 kHz, SE increases with ultrasonic power to a maximum value and then decreases. At 305 kHz, SE increases with ultrasonic power and then reaches two peaks before decreasing. At 400, 514, 1018, and 1960 kHz, SE increases with ultrasonic power, becomes nearly constant, and then decreases significantly. The maximum SE at an ultrasonic power of 5.9 W and frequency of 209 kHz is 16.4 × 10^−10^ mol·J^−1^, which is approximately twice the value of 8.3 × 10^−10^ mol·J^−1^ at 200 kHz that is reported by Koda et al. [30].

**Fig. 4:**
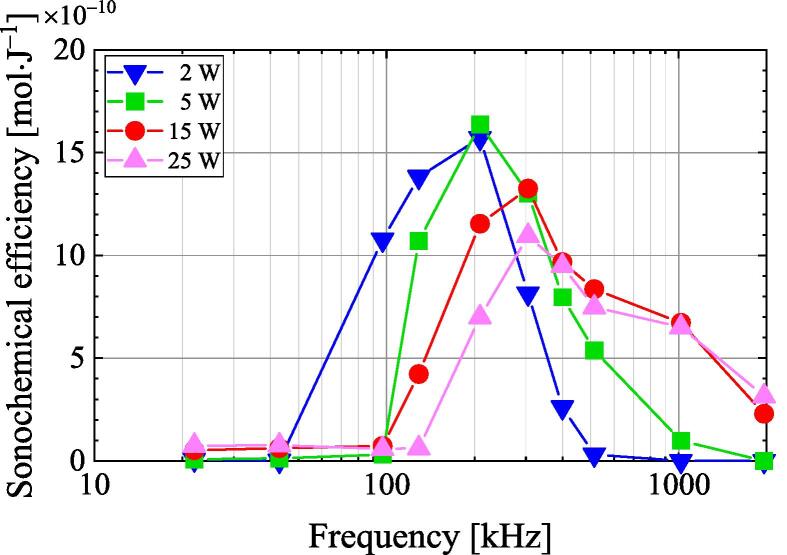
Plot of sonochemical efficiency against frequency at four discrete ultrasonic powers.

Fig. 4 plots SE against frequency at four different ultrasonic powers. The sample volume is 100 mL. SE increases with ultrasonic frequency before reaching a maximum value and then decreases. The relationship between SE and frequency is affected by ultrasonic power. The dependence of the SE on the frequency at an ultrasonic power of 15 W is similar to that reported by Koda et al. [30]. The peak SE occurs at lower frequencies when the ultrasonic power is decreased.

#### 3.2.2 Dependence of the reaction rate on ultrasonic power and frequency

**Fig. 5:**
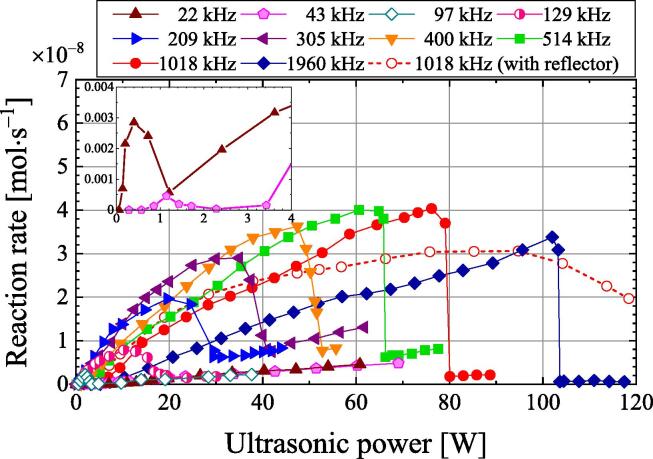
Dependence of the I_3_^−^ reaction rate on ultrasonic power at various frequencies (inset: magnified reaction rates at 22 and 43 kHz).

Fig. 5 shows the I_3_^−^ reaction rate as calculated with Eq. (4). The reaction rate is defined as the change in concentration per unit time and is equal to the product of SE multiplied by the ultrasonic power. The inset shows the magnified reaction rates at frequencies of 22 and 43 kHz. At all frequencies, the reaction rate increases with the ultrasonic power, reaches a maximum, and then decreases significantly. The maximum reaction rate increases with frequency, except for 1960 kHz. The maximum reaction rate at a frequency of 1018 kHz is 4.0 × 10^−8^ mol·s^−1^ at an ultrasonic power of 76 W. This is twice the maximum reaction rate at a frequency of 209 kHz, which is 2.0 × 10^−8^ mol·s^−1^ at an ultrasonic power of 20 W. The reaction rate decreases significantly once the ultrasonic power is increased beyond the value at the maximum reaction rate. The minimum reaction rate is observed at ultrasonic powers and frequencies of 66 W at 514 kHz, 80 W at 1018 kHz, and 104 W at 1960 kHz. Increasing the ultrasonic power after the minimum reaction rate is reached caused the reaction rate to increase slightly. The decrease in the reaction rate despite an increase in ultrasonic power is called quenching [38]. Increasing the frequency causes quenching to occur at a higher ultrasonic power.

When ultrasound is applied to water by a transducer, a traveling wave propagates from the transducer toward the water surface or reflector. The traveling wave is then reflected by the water surface or reflector back to the transducer. If the traveling and reflected waves propagate without attenuation, only a standing wave is generated by the interference between the two waves. In reality, however, the traveling and reflected waves gradually attenuate during propagation. The attenuation of ultrasound increases with frequency. When the reflected wave is weaker than the traveling wave because of attenuation, traveling wave components appear in addition to the standing wave in the sound field. The traveling wave component is expressed as the difference between the traveling and reflected waves. The traveling wave component that propagates from the transducer to the water surface or reflector is superimposed on the standing wave of the sound field.

Cavitation occurs when bubbles collapse. The bubbles receive both a radiation force due to the traveling wave component from the transducer to the water surface or reflector and the primary Bjerknes force, which is a type of radiation force generated in a standing wave [40], [41], [42], [43]. When the difference between the traveling and reflected waves is small, the primary Bjerknes force is greater than the radiation force due to the traveling wave component. Therefore, many bubbles are trapped in the antinode of the sound pressure of the standing wave by the primary Bjerknes force. Such a sound field is defined as a standing wave field. However, if the reflected wave is smaller than the traveling wave and the radiation force due to the traveling wave component is greater than the primary Bjerknes force, few bubbles are trapped in the standing wave, and they instead move from the transducer toward the water surface or reflector. Such a sound field is defined as a traveling wave field. Near the water surface or reflector, the standing wave field forms because the traveling wave and reflected wave are almost the same in strength. As a result, a chemical reaction field is formed by bubbles trapped in the standing wave. However, near the transducer, the traveling wave field is formed because the reflected wave is smaller than the traveling wave. Therefore, the bubbles move toward the water surface, and a small chemical reaction field is formed [44]. The authors previously investigated the sonochemical reaction field at 129 and 490 kHz using sonochemical luminescence and reported that the sonochemical reaction field was mainly observed near the water surface [31]. At these high frequencies, the sonochemical reaction field forms in a standing wave near the water surface. Fig. 5 shows the ultrasonic power dependence of the reaction rate at 1018 kHz with a stainless steel reflector on the water surface (red open circles and dotted line). The reaction rate does not drop with increasing ultrasonic power after the maximum reaction rate is reached but decreases gradually. In other words, a gradual quenching phenomenon is observed. Quenching occurs with increasing ultrasonic power beyond the maximum chemical reaction rate because the traveling wave field increases and standing wave field decreases with an increasing ultrasonic power. This reduces the chemical reaction field. Furthermore, the expansion and the contraction of bubbles produces a phase difference because of the higher sound pressure. As a result, bubbles trap in the antinodes of the sound pressure of the standing wave by the primary Bjerknes force are repelled [40], and the number of bubbles decreases. When two bubbles expand and contract in the same phase, they are attracted by the secondary Bjerknes force, which is a kind of radiation force [39], [40]. As the sound pressure and secondary Bjerknes force increase, the bubbles aggregate or coalesce, and they become larger and no longer contribute to the chemical reaction. Without a reflector, quenching occurs due to reduction of the standing wave field and the reduction of bubbles, just as the case with a reflector. Without a reflector, the water surface is free and moves significantly because of the radiation force at high ultrasonic powers. This causes the standing wave field to move significantly as well. Because of the violent movement of the standing wave field near the water surface, bubbles are not trapped in the unstable standing wave, and sudden quenching occurs [45]. By contrast, the chemical reaction rate decreases gradually with the reflector. Because the standing wave field is stable and does not move in this case, sudden quenching does not occur.

Cavitation bubbles are generated by the growth of bubble nuclei induced by ultrasonic irradiation. This causes degassing, which reduces the amount of dissolved gas in the water. In this study, degassing occurs below the ultrasonic power at which sudden quenching occurs, and almost no degassing occurs at higher ultrasonic powers except at 22 and 43 kHz. When the ultrasonic power was below the point at which sudden quenching occurred, many bubbles were observed on the sides of the vessel. However, except at 22 and 43 kHz, no bubbles were observed when the ultrasonic power was above the point at which sudden quenching occurred. Therefore, when sudden quenching occurs, almost no bubbles grow from the bubble nuclei except at 22 and 43 kHz. Hatanaka et al. reported that bubble clusters were observed at 23 kHz when quenching occurred [28]. When quenching occurs, the clustering of bubbles or the disappearance of bubbles may be related to the frequency. At ultrasonic powers of 50–80 W and a frequency of 1018 kHz, the chemical reaction rate is higher without the reflector than with the reflector. In the absence of the reflector, the number of cavitation bubbles increases and the sonochemical reaction rate increases because of the transport of gas between the gas and liquid phases.

In the frequency range of 129–1960 kHz, the ultrasonic power at which sudden quenching occurs (Fig. 5) is the same as the ultrasonic power at which a sudden decrease is observed with increasing electric power (Fig. 2). This indicates that the sudden decrease in ultrasonic power can be attributed to the sudden quenching with no reflector. When the sudden quenching occurs, the number of bubbles suddenly decreases and the heat in the sample due to the vibration of the bubbles suddenly decreases. Then, the reflected wave from the water surface increases in strength because of the reduced ultrasound attenuation and scattering by the bubbles. The transmitted wave in the transducer increases, and the heat dissipation in the transducer increases. Therefore, the heat in the sample is suddenly reduced by the sudden quenching. For cases with low power and without sudden quenching, the authors assume that there are more bubbles near the water surface without a reflector than with a reflector. Thus, more heat is generated by the vibration of bubbles without a reflector than with a reflector. This explains why the ultrasonic power is greater without the reflector than with the reflector at a frequency of 1018 kHz and electric power of less than 130 W (Fig. 2).

The primary Bjerknes force is proportional to the frequency and bubble volume. The bubble radius at which a bubble collapses during cavitation is approximately inversely proportional to the frequency. Therefore, the primary Bjerknes force increases with decreasing frequency. Because the secondary Bjerknes force is also proportional to the bubble volume, it also increases with decreasing frequency. Thus, the Bjerknes forces should increase and the ultrasonic power at which quenching occurs should decrease with decreasing frequency.

**Fig. 6:**
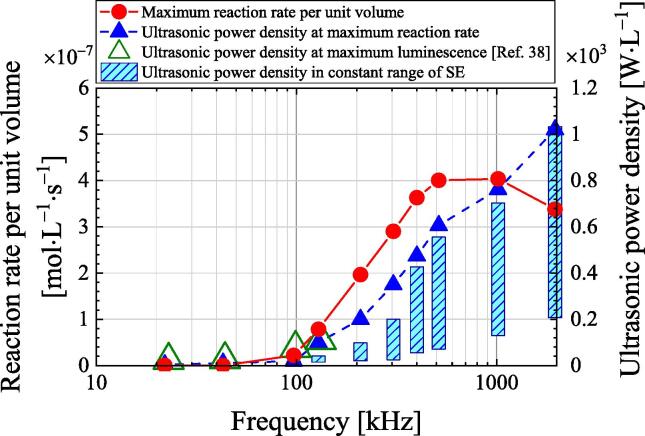
Frequency dependence of the maximum reaction rate per unit volume and the ultrasonic power density at the maximum reaction rate.

The frequency dependence of quenching is evaluated according to the ultrasonic power density (i.e., ultrasonic power divided by the volume) and reaction rate per unit volume (i.e., reaction rate divided by the volume). Fig. 6 plots the maximum reaction rate per unit volume and corresponding ultrasonic power density against the frequency. These values are obtained by dividing the data in Fig. 5 by the sample volume of 100 mL. Hatanaka et al. [38] irradiated a sample with a volume of 1 L with ultrasound at frequencies of 23, 44, 99, and 132.2 kHz to investigate the sonoluminescence of multiple bubbles using a photomultiplier tube, and they reported the quenching phenomenon. The sonoluminescence intensity reaches its maximum at electric powers of 57, 66, 186, and 273 W and frequencies of 23, 44, 99, and 132.2 kHz, respectively. The ultrasonic power is then calculated under the assumption that the transducers had an efficiency of 40%. The ultrasonic power densities for a sample volume of 1 L are also plotted in Fig. 6. In terms of the ultrasonic power density, the experimental results of Hatanaka et al. are in close agreement with the results of this study. Therefore, the ultrasonic power at which quenching occurs can be roughly estimated for different sample volumes.

The bars in Fig. 6 show the range of ultrasonic power densities at which the SE is above 80% of the maximum SE (Fig. 3) for the corresponding frequency. Within these ranges of the ultrasonic power density, the SE is almost constant regardless of the ultrasonic power density. The ultrasonic power density range increases with the frequency.

#### 3.2.3 Dependence of the reaction rate on the sample volume

**Fig. 7:**
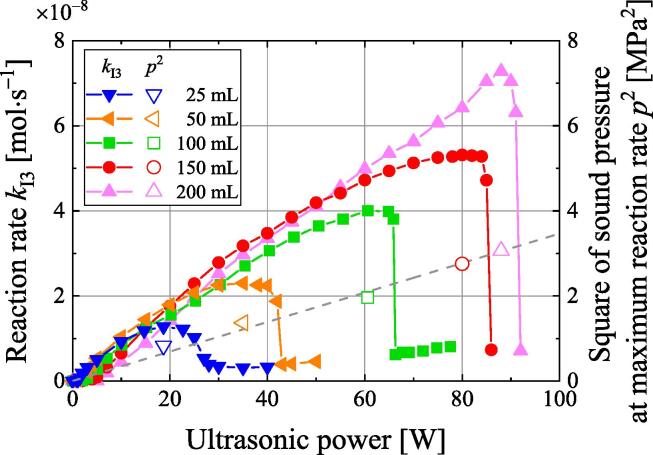
Relationship between the reaction rate and sound pressure squared to the ultrasonic power for five sample volumes.

The dependence of the reaction rate on the sample volume was investigated. Fig. 7 shows the relationship between the ultrasonic power and the reaction rate at a frequency of 514 kHz for sample volumes of 25, 50, 100, 150, and 200 mL. The relationship between the ultrasonic power and reaction rate shows strong dependence on the sample volume, and quenching occurs for all sample volumes. The ultrasonic power at which quenching occurs increases with the sample volume. The volume dependence of the quenching shows the same trend as the fall off at 1 MHz reported by Henglein et al [46]. For small sample volumes (e.g., 25 mL), the reaction rate is greatly reduced by quenching at low ultrasonic powers (e.g., 30 W).

Fig. 7 also shows the square of the sound pressure in the sample at the ultrasonic power with the maximum reaction rate. The sound pressure was measured by placing a hydrophone (HUS-200S, Honda Electronics) at the position with the maximum sound pressure for each volume. The square of the sound pressure is approximately proportional to the ultrasonic power. The sound pressure at which quenching occurs increases with the sample volume. Thus, the sound pressure at which quenching occurred was not constant and depended on the volume. It was found that quenching does not have a sound pressure threshold, which is a constant sound pressure to initiate quenching.

**Fig. 8:**
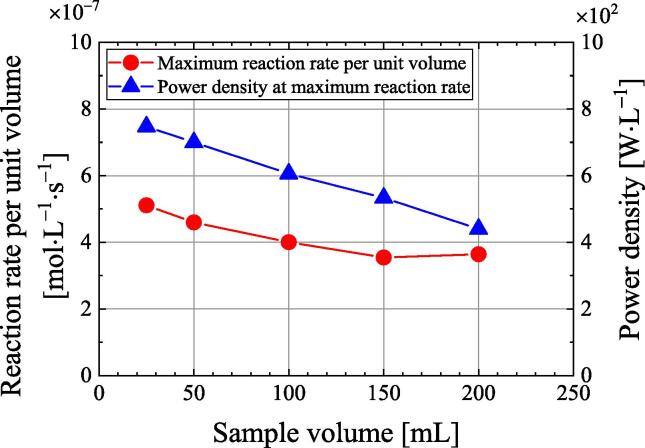
Dependence of the reaction rate per unit volume and ultrasonic power density on the sample volume.

Fig. 8 plots the maximum reaction rate per unit volume and the corresponding ultrasonic power density against the sample volume. These values are obtained by dividing the data in Fig. 7 by the sample volume. The maximum reaction rate per unit volume decreases slightly as the sample volume increases. The reaction rate decreases with increasing sample volume (i.e., increasing liquid height) because the chemical reaction field is not uniform [31]. As the sample volume increases, the ultrasonic power density at the maximum reaction rate decreases. The ultrasonic power density at which quenching occurs is not constant with respect to the sample volume. This is because the ultrasonic power density at which quenching occurs may also depend on the liquid height, the inner diameter and the shape of the vessel, the sound field such as the near field, and other factors. The dependence of the SE on the frequency [30] and liquid height [31] seems to be strongly related to the quenching phenomenon. Such topics will be researched in the near future.

## 4 Conclusions

Ultrasound was directly irradiated to a sample volume of 100 mL in a vessel with an inner diameter of 56 mm, and the relationship between the ultrasonic power and reaction rate was investigated for the frequency range of 22–1960 kHz. The effect of the sample volume from 25 to 200 mL was also evaluated at a frequency of 514 kHz. The reaction rate was determined with the KI method. Quenching (i.e., a decrease in the reaction rate despite an increase in ultrasonic power) was observed at all frequencies and sample volumes. The maximum reaction rate increased with the frequency and sample volume, except at 1960 kHz. The ultrasonic power at which quenching occurred increased with the frequency and sample volume. The quenching phenomenon was affected by the presence of a reflector on the water surface. Quenching was gradual with the reflector and sudden without it. The results showed that ultrasonic power density can be used to roughly estimate the ultrasonic power at which quenching occurs. In addition, the ultrasonic power density range with a high reaction efficiency and no quenching was clarified. To develop a highly efficient sonochemical reactor, a standing wave field with a stable and large volume should be formed, and gas should be supplied to this reaction field.

### CRediT authorship contribution statement

**Yoshiyuki Asakura:** Conceptualization, Methodology, Software, Validation, Formal analysis, Investigation, Resources, Data curation, Writing – original draft, Writing – review & editing, Visualization, Supervision, Project administration. **Keiji Yasuda:** Validation, Resources, Writing – review & editing, Funding acquisition.

## Declaration of Competing Interest

The authors declare that they have no known competing financial interests or personal relationships that could have appeared to influence the work reported in this paper.
